# Efficient Design of Compact Unstructured RNA Libraries Covering All *k*-mers

**DOI:** 10.1089/cmb.2015.0179

**Published:** 2016-02-01

**Authors:** Yaron Orenstein, Bonnie Berger

**Affiliations:** ^1^ Computer Science and Artificial Intelligence Laboratory, Massachusetts Institute of Technology, Cambridge, MA.; ^2^ Department of Mathematics, Massachusetts Institute of Technology, Cambridge, MA.

**Keywords:** de Bruijn graph, microarray library design, RNA secondary structure

## Abstract

**Current microarray technologies to determine RNA structure or measure protein–RNA interactions rely on single-stranded, unstructured RNA probes on a chip covering together all *k*-mers. Since space on the array is limited, the problem is to efficiently design a compact library of unstructured *ℓ*-long RNA probes, where each *k*-mer is covered at least *p* times. Ray et al. designed such a library for specific values of *k*, *ℓ*, and *p* using ad-hoc rules. To our knowledge, there is no general method to date to solve this problem. Here, we address the problem of finding a minimum-size covering of all *k*-mers by *ℓ*-long sequences with the desired properties for any value of *k*, *ℓ,* and *p*. As we prove that the problem is NP-hard, we give two solutions: the first is a greedy algorithm with a logarithmic approximation ratio; the second, a heuristic greedy approach based on random walks in de Bruijn graphs. The heuristic algorithm works well in practice and produces a library of unstructured RNA probes that is only ∼1.1-times greater in size compared to the theoretical lower bound. We present results for typical values of *k* and probe lengths *ℓ* and show that our algorithm generates a library that is significantly smaller than the library of Ray et al.; moreover, we show that our algorithm outperforms naive methods. Our approach can be generalized and extended to generate RNA or DNA oligo libraries with other desired properties. The software is freely available online.**

## 1. Introduction

RNAs play vital roles in many processes in the living cell. Through interaction of RNAs with other RNAs or proteins, they perform specific functions. RNA–RNA interactions play a role in many pathways of RNA metabolism, including pre-mRNA splicing, ribosome synthesis, and the regulation of mRNA stability by microRNAs (Kudla et al., 2011). RNA-binding proteins interact with RNAs to modulate and affect a wide variety of cellular processes, including RNA replication, repair, and recombination (Rinn and Ule, 2014). Both types of interactions are mediated through the structure and sequence of the RNA molecule. Typically, interactions occur with RNA accessible regions through either base-pairing to nucleotides of another RNA or hydrogen bonding to a protein's residues (Wan et al., 2011; Kertesz et al., 2007).

A given RNA may fold into different conformations, which vary in accessible regions (Steffen et al., 2006); therefore, relying on *in silico* prediction of its structure may lead to incorrect predictions for the accessible region of interest. Researchers would thus like to experimentally measure accessible regions in RNAs.

Numerous experimental methods have been developed to study the secondary structure of RNAs in a high-throughput (HTP) manner (Kertesz et al., 2010; Mandir et al., 2009; Kierzek et al., 2006). Microarray technologies measure RNA secondary structure through the hybridization of accessible regions to a set of oligos on a chip. An array covering all RNA *k*-mers (a contiguous RNA word of length *k*) can robustly and accurately measure the structure of many RNAs. Examples for such arrays covering all 6-mers and 7-mers include a couple of studies (Mandir et al., 2009; Kierzek et al., 2006), respectively. In both experimental setups, each oligo contains a unique *k*-mer. Despite the fact that microarrays are limited in throughput compared to deep-sequencing-based methods, they are still often being used to overcome limitations in sequencing methods (Kierzek et al., 2015).

RNA-binding proteins (RBPs) regulate gene translation post-transcriptionally via their binding to RNA molecules. More than 1,500 genes in the human genome are thought to code for RBPs, making this family one of the largest families in the human proteome (Gerstberger et al., 2014). Many of these proteins have sequence-specific RNA-binding properties and thus regulate genes by binding only to site-specific elements. Better characterization of RBP's sequence-specific binding preferences can improve our understanding of post-transcriptional gene regulation.

New experimental high-throughput (HTP) techniques have been developed to uncover protein–RNA interactions on a genome-wide scale at single-nucleotide resolution. For example, HITS-CLIP, CLIP-seq, and RIP-seq (König et al., 2012) measure protein–RNA interactions *in vivo* in an HTP manner. However, much like protein DNA-binding, protein RNA-binding is influenced by a variety of factors, such as other RBPs (that either compete for the same binding site or cobind as a complex) and RNA secondary structure, which determines if a binding site is accessible or not (Fu and Ares Jr. 2014). While the end goal is to understand and predict *in vivo* binding, *in vitro* experiments currently have higher resolution and lower noise and thus provide valuable complementary information to protein RNA-binding preferences.

Toward this aim, high-throughput *in vitro* methods have been developed to study the binding preferences of RBPs (Ray et al., 2009; Lambert et al., 2014). In RNAcompete (Ray et al., 2009), a specific protein binds to a set of predesigned oligos, and binding is measured using a florescence tag. The binding of the protein to a set of more than 200,000 probe sequences is reported. A recent study by the authors presents the binding of more than 200 human RBPs and provides a compendium of RBPs (Ray et al., 2013). RNA Bind-n-Seq is a new technology that measures protein RNA-binding based on HTP sequencing (Lambert et al., 2014). Since the initial library is composed of random oligos, these may be structured and as a result include *k*-mers that are likely to be base-paired in RNA secondary structure.

The oligo library used in RNAcompete experiments has unique properties that allow it to effectively measure protein RNA-binding in a universal and unbiased manner. The complete oligo set is designed such that each 9-mer is covered at least 16 times. This property guarantees the ability to infer accurate binding scores for 9-mers and shorter *k*-mers. Another key property is that the probe sequences are unstructured, which makes them accessible to the protein for binding (Stefl et al., 2005).

In this article, we address the problem of designing better microarray probe libraries for enhanced exploration of RNA structure through base-pairing of the target RNA to the probes as well as protein RNA-binding through affinity between a protein and the probes. Note that array designs that consist of a single *k*-mer for each probe are disadvantageous: the space on the microarray is limited, while the number of probes grows exponentially with *k* (the number of possible RNA *k*-mers is 4^*k*^). A small value of *k* is also undesirable, since the likelihood of having a *k*-mer appear more than once in a target RNA sequence, and thus preventing unique identification of accessible sites, increases as *k* gets smaller. Hence, we aim to increase the size of *k*, while maintaining a small number of oligos on the chip. This goal can be achieved by covering a number of *k*-mers on each oligo. In this scenario, the *k*-mers are no longer covered by a unique sequence. Alternatively, if a *k*-mer is covered multiple times, an aggregate score for its accessibility or affinity can be inferred.

There are numerous methods to design sequences with complete coverage of all *k*-mers. De Bruijn sequences are the most compact sequences to cover all *k*-mers (Berger et al., 2013). They can be generated in linear time in various ways, including Euler tours in complete de Bruijn graphs (West et al., 2001), linear-feedback shift registers (Lempel, 1970), and in a recursive manner (Alhakim and Akinwande, 2011). De Bruijn sequences have been successfully used in HTP technologies that measure protein DNA-binding, such as protein-binding microarrays (Berger et al., 2006; Philippakis et al., 2008; Orenstein and Shamir, 2013), and MITOMI (Fordyce et al., 2010).

However, the coverage of all *k*-mers is not enough, as RNAs may form structure. In RNAcompete, the authors used ad-hoc greedy rules to generate an oligo library with the desired properties (Ray et al., 2009); however, their method cannot be generalized. To our knowledge, there is currently no method to generate an RNA oligo library such that each *k*-mer occurs at least *p* times in *ℓ*-long unstructured probe sequences. Such a method would be highly useful for current and future technologies that measure protein–RNA interactions or RNA secondary structure. In addition, the freed space on the device may be used to cover longer *k*-mers or sequences with other specific properties.

Here, we solve the problem of designing an RNA oligo library such that each *k*-mer occurs at least *p*-times in *ℓ*-long unstructured probe sequences. We prove that for a given set of *ℓ*-long probes, the problem of covering all *k*-mers by a minimum-size subset is NP-hard. Thus, we formulate the problem as a minimum *m*-set cover problem and give an approximation algorithm with guaranteed logarithmic ratio. We also present a heuristic greedy algorithm based on random walks in de Bruijn graphs, which perform very well in practice; it produces an oligo library that is only ∼1.1-times greater in size than the theoretical lower bound. In our results, we analyze the fraction of unstructured RNA oligos as a function of their length and show that traditional methods to cover all *k*-mers do not work. We conclude with an analysis of the computational performance of our heuristic algorithm over different values of *k* and *ℓ* and in comparison to the design of RNAcompete (Ray et al., 2013). The software is freely available online.

## 2. Preliminaries

### 2.1. de Bruijn graphs

A *de Bruijn graph* of order *k* over alphabet *Σ* is a directed graph in which every vertex has an associated label (a string over *Σ*) of length *k* (*k*-mer) and every edge has an associated label of length *k* + 1. There are exactly |*Σ*|^*k*^ vertices in the graph, each representing a unique *k*-mer. If an edge (*u*, *v*) has an associated label *l*, then the label associated with *u* must be a *k*-prefix of *l*, and the label associated with *v* must be a *k*-suffix of *l*. A complete de Bruijn graph contains all possible edges, which represent together all (*k* + 1)-mers over *Σ*.

Every path in a de Bruijn graph represents a sequence. A path *v*_1_*,e*_1_*,v*_2_*,…,v_n_* of length *n* spells a sequence *s* of length *n* + *k* − 1 such that the label associated with *v_i_* occurs in *s* at position *i* for all 1 ≤ *i* ≤ *n*, and the label associated with *e_i_* occurs in *s* at position *i* for all 1 ≤ *i* ≤ *n* − 1.

### 2.2. Unstructured RNA probes and self-structured k-mers

We followed the definition of *structuredness* used in the RNAcompete study (Ray et al. 2009). The authors use RNAshapes (Steffen et al., 2006) to enumerate all secondary structures with free energies within 70% of the minimum free energy. The exact command line is: RNAshapes -s -c 70.0 -r -M 30 -t 1 -o 2.

The sum of the probabilities of structures with free energies less than −2.5 kcal/mol quantifies structuredness. A value below 0.5 is considered *unstructured*. For any sequence, we prepend the linker used in the RNAcompete technology (*AGG* or *AGA*) (Ray et al., 2009). From the two linkers, we selected the one that gave the smaller sum of probabilities.

A *self-structured k*-mer forms structure in itself. It follows that no probe can contain it without being structured. Thus, to cover all *k*-mers in a microarray, structured probes must be included. For the structure definition above, self-structured *k*-mers exist for *k* ≥ 9. Smaller values of *k* do not require structured probes to cover all *k*-mers. We refer to *k*-mers that are not self-structured as *unstructured k*-mers.

### 2.3. Problems definition

We first define the notion of *k-mer coverage* over alphabet *Σ*.

**Definition 1.** *A set L of sequences is a k-mer coverage over Σ if for every w* ∈ *Σ^k^, there exists a sequence L_i_*∈*L s.t. w*∈*L_i_.*

We generalize the definition of *k*-mer coverage with a *p-multi k-mer coverage*.

**Definition 2.** *A set L of sequences is a p-multi k-mer coverage over Σ if for every w* ∈ *Σ^k^,* $\sum \nolimits_{L_i \in L} {o ( w , L_i ) } \geq p$*, where o*(*w*, *L_i_*) *is the number of times w occurs in sequence L_i_.*

We can now state our optimization problem:

**THE MINIMUM** *K***-MER COVERAGE BY** *ℓ***-LONG SEQUENCES PROBLEM**

INSTANCE: A set *S* of *ℓ*-long sequences that is a *k*-mer coverage over *Σ* = {*A*, *C*, *G*, *U*}.

VALID SOLUTION: A subset *S*′ ⊆ *S* that is a *k*-mer coverage over *Σ*.

GOAL: Minimize |*S*′|.

And a similar NP-hard problem that we reduce from and use for an approximation algorithm:

**THE MINIMUM** *M***-SET COVER PROBLEM**

INSTANCE: A set *S* of subsets of *E* = {*e*_1_,…,*e_n_*} s.t. for any *S_i_*∈*S*, its size |*S_i_*|≤ *m*.

VALID SOLUTION: A subset *S*′ ⊆ *S* s.t. for every *e_i_*∈*E* there exists *S_i_*∈*S*′ s.t. *e_i_*∈*S_i_*.

GOAL: Minimize |*S*′|.

We generalize the *k*-mer coverage problem by requiring multiple *k*-mer occurrences. Note that multisets may contain an element multiple times. We use *distinct*(*S*) to denote the set of unique elements in multiset *S*.

**THE MINIMUM** *P***-MULTI** *K***-MER COVERAGE BY** *ℓ***-LONG SEQUENCES PROBLEM**

INSTANCE: A set *S* of *ℓ*-long sequences that is a *k*-mer coverage over *Σ* = {*A*, *C*, *G*, *U*} and *p*.

VALID SOLUTION: A multiset *S*′ s.t. *distinct*(*S*′) ⊆ *S* and *S*′ is a *p*-multi *k*-mer coverage over *Σ*.

GOAL: Minimize |*S*′|.

**Dealing with self-structured** ***k*-mers** Note that since self-structured *k*-mers may exist, covering all *k*-mers by *ℓ*-long unstructured probes may be impossible. The coverage problem may be redefined as two subproblems to handle self-structured *k*-mers:

1. Cover all **unstructured** *k*-mers by a minimum size set of *ℓ*-long unstructured RNA probes.
2. Cover all **self-structured** *k*-mers by a minimum size set of *ℓ*-long RNA probes.

The union of these sets covers all *k*-mers, since each *k*-mer is either unstructured or self-structured by definition (see section 2.2).

## 3. Methods

Since the minimum *k*-mer coverage by *ℓ*-long sequences problem is NP-hard (as we prove in section 3.3), we provide an approximation algorithm and heuristic, which performs very well in practice, to address this problem.

### 3.1. Approximation algorithm through the minimum *m*-set cover problem

The problem of covering all *k*-mers in unstructured RNA probes can be formulated as a minimum *m*-set cover problem. The problem can be approximately solved by a greedy algorithm. The algorithm starts with an empty set and adds to the solution the set that has the most uncovered elements in it. The algorithm achieves an approximation ratio of $H ( m ) - \frac { 196 }  { 360 }$, where *m* is the maximum cardinality of a set in *S*, and *H* is the harmonic number $H ( n ) = \sum \nolimits_{i = 1}^n {1 / i} \leq ln ( n ) + 1$ (Berman et al., 2004; Levin, 2008). The algorithm can be highly accurate in some instances (Grossman and Wool, 1997). This leads us to the next corollary:

**Corollary 1.** *Algorithm 1 is an* ($H_ { \ell - k + 1 } - \frac { 196 }  { 390 }$)*-approximation to the minimum k-mer coverage by ℓ-long sequences problem.*

If self-structured *k*-mers exist, Algorithm 1 can be modified to first handle the coverage of unstructured *k*-mers by unstructured RNA probes, and then rerun to cover uncovered self-structure *k*-mers by structured RNA probes. Thus, since the approximation ratio is valid for each subproblem, Corollary 1 is valid for covering all *k*-mers (see section 2.3 for definition of subproblems).

**Algorithm 1:** Solve *k*-coverage by *ℓ*-long unstructured RNA probes problem as a set cover problem

1: For each *ℓ*-long RNA sequence:
2: Test if the sequence is unstructured. If so, add it to the list of unstructured sequences.
3: Apply the greedy set cover algorithm:
4: The elements are the *k*-mers.
5: The sets are the unstructured sequences and their elements are the *k*-mers they cover.

The running time of the algorithm is exponential in the oligo length *ℓ*. The first step iterates over all possible *ℓ*-long sequences, and for each one runs an RNA secondary structure prediction algorithm. Denote *f* (*ℓ*) to be the running time of the prediction algorithm on an *ℓ*-long sequence; then Step 1 takes *Θ*(4^*ℓ*^ · *f* (*ℓ*)). The second step can be implemented using a priority queue, whose keys are the number of uncovered elements of each unstructured sequence not in the solution. Since the keys are integers bounded by *ℓ*−*k* + 1, queue operations can be implemented in *O*(1) time. A dictionary is used to hold uncovered *k*-mers and pointers to the sequences that contain them. The dictionary can be implemented using an array of size 4^*k*^, as our *k*-mers can be represented as integers from 0 to 4^*k*^ − 1. Each cell contains a list of pointers to sequences containing the *k*-mer. The length of the list is bounded by (*ℓ* − *k* + 1) · 4^*ℓ*-*k*^, the number of possible *ℓ*-long sequences containing the *k*-mer. Therefore, we get that the running time for the second step is *Θ*(4^*ℓ*^ + 4^*k*^ · (*ℓ − k* + 1) · 4^*ℓ*−*k*^). The first term consists of delete-minimum operations on the queue, and the second, the update operations. In total, Algorithm 1 takes time *Θ*(4^*ℓ*^ · *f* (*ℓ*)). [Since input size is *ℓ*, then *f* (*ℓ*) = *Ω*(*ℓ*). Predicting minimum free-energy structure can be done in *O*(*ℓ*^2^) (Lorenz et al., 2011). Predicting all possible structures takes *O*(4^*ℓ*^ · *ℓ*) as there is an exponential number of structures, and heuristics are used to estimate representative structures up to a given energy threshold (Steffen et al., 2006)]. Unfortunately, the running time is infeasible for most instances, for example, *ℓ* = 35 in RNAcompete's implementation (Ray et al., 2009). Thus, we turn to a heuristic greedy algorithm.

### 3.2. A heuristic greedy algorithm based on random walks in de Bruijn graphs

Our greedy algorithm, summarized as Algorithm 2, is based on the following two key ideas:

1. Using random walks in a de Bruijn graph to find unstructured oligos.
2. Backtracking strategy in cases where the random walk reaches a structured oligo.

**FIG. 1.**
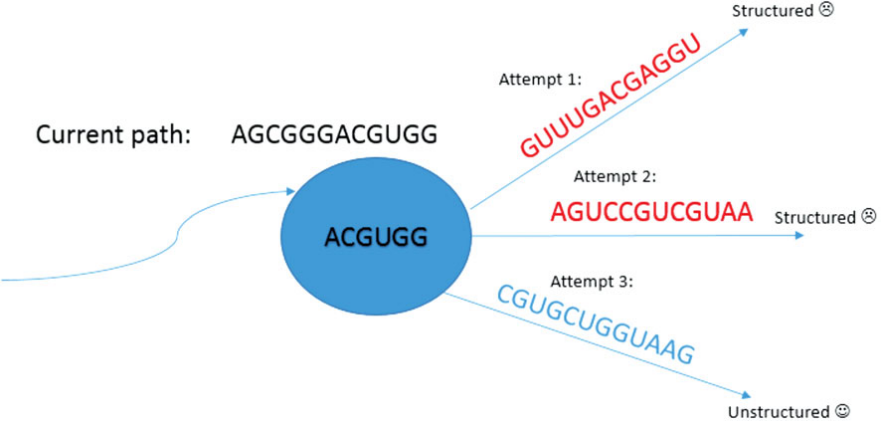
An illustration of the search process for unstructured paths. In the example, the current path started from vertex *AGCGGG*. It was extended to the unstructured path *AGCGGGACGUGG*. Then, it attempted to extend the path and succeeded in the third attempt to find an unstructured path. The de Bruijn graph is of order 6 to cover all 7-mers.

The algorithm tries to find a set of disjoint *ℓ*-long paths in a de Bruijn graph, each representing an unstructured probe, and together covering all the edges. To cover each *k*-mer *p* times, *p* − 1 copies are added to each edge. During the search for the desired paths, structured paths may be found. To address this problem, the algorithm backtracks and searches for a different path. An illustration of this process is depicted in Figure 1. Through its random walk, the algorithm doubles the length of the explored path by possible extensions and selects the first unstructured path it encounters. The rationale behind this search process follows from two ideas related to RNA secondary structure prediction:

1. If a subsequence is structured, it is most likely that a sequence containing it is structured (for experimental support see section 4.1).
2. A structure may form between one half of a sequence to the other half.

Thus, the algorithm does not waste time by trying to extend structured subpaths. Indeed, it considers all possible path-extensions of length double the current path to test if it is unstructured. This fact is also beneficial in terms of running time: the number of extensions in a doubling scheme is *O*(log(*ℓ*)) instead of *O*(*ℓ*).

**Algorithm 2:** Generate a set of *ℓ*-long unstructured RNA sequences covering all *k*-mers *p* times. Input: *k* (coverage), *ℓ* (oligo length), *p* (multiplicity), *c* (a limit on the number of attempts)

1: Generate a complete de Bruijn graph of order *k* − 1. For each edge add *p* − 1 copies.
2: Initialize a list *L* of unfinished vertices with all vertices.
3: Set *current*_*vertex* to the first element in the list.
4: **while** there are edges in the graph **do**
5: *probe* = label of *current*_*vertex*.
6: *extension*_*length* = *ℓ*.
7: **while** |*probe*| *< extension*_*length***do**
8: Try to extend probe to length *minimum*{2 · |*probe*|, *extension*_*length*}.
9: **if** unstructured extension was not found in *c* attempts **then**
10: *extension*_*length* = *extension*_*length* − 1.
11: **end if**
12: **end while**
13: **if** |*probe*| = *k* − 1 **then**
14: Extend *probe* by a random extension of size 1.
15: **end if**
16: Output probe and delete the edges of its *k*-mers from the graph.
17: **if***current*_*vertex* has no outgoing edges AND |*L*| *>* 1 **then**
18: remove it from *L*.
19: Set *current*_*vertex* to a random vertex from *L*.
20: **end if**
21:** end while**

We bound the running time of the algorithm. The number of possible extensions at each vertex is at most 4^*i*^, where *i* is the length of the current probe. Since the maximum number of extensions at any vertex is $4^{\lfloor \ell / 2 \rfloor}$, the sum of possible extensions examined for each probe is *Θ*(4^*ℓ/*2^). Denote by *f* (*ℓ*) the running time of the prediction algorithm and the number of probes by *X*, then the total time is *O*(*X*4^*ℓ/*2^*f* (*ℓ*)). This may be prohibitive in some instances, depending on the value of *ℓ*. Thus, for practical reasons, we replace the search of all possible extensions by a search of a limited number of random extensions. Denote this number *c* (given as input) and remember that the extensions are performed in a doubling scheme. Hence, the total running time is *O*(*Xf* (*ℓ*)*c*log(*ℓ*)). Results show that *X* = *Θ*(4*^k^/*(*ℓ* − *k* + 1)) (see Table 1).

**Table 1.** Computational Results for Different Oligos Libraries

ℓ	k	*Lower bound*	*Incomplete set*	*Incomplete ratio*	*Complete set*	*Complete ratio*	*Structured*	*Naive set*	*Runtime (hh:mm:ss)*
30	5	40	50	1.25	51	1.27	0	149	00:02:11
	6	164	182	1.11	182	1.11	0	766	00:07:43
	7	684	737	1.08	739	1.08	0	3,308	00:41:40
	8	2,850	3,081	1.08	3,106	1.09	0	13,801	02:58:52
	9	11,916	12,940	1.09	13,069	1.10	59	57,154	14:42:27
	10	49,934	55,882	1.12	56,526	1.13	670	236,477	82:18:01
35	5	34	41	1.21	41	1.21	0	131	00:03:13
	6	138	158	1.14	162	1.17	0	670	00:21:20
	7	566	635	1.12	648	1.15	0	2,884	01:17:43
	8	2,342	2,670	1.14	2,744	1.17	0	11,961	06:03:05
	9	9,710	11,022	1.14	11,439	1.18	60	49,289	26:47:31
	10	40,330	47,139	1.17	49,225	1.22	609	202,763	137:33:27
40	5	30	37	1.23	38	1.27	0	117	00:02:44
	6	118	140	1.19	148	1.25	0	598	00:36:31
	7	482	561	1.16	611	1.27	0	2,561	02:33:16
	8	1,986	2,362	1.19	2,627	1.32	0	10,597	11:24:15
	9	8,192	9,745	1.19	10,966	1.34	60	43,492	48:02:15
	10	33,826	41,798	1.24	47,457	1.40	557	178,187	246:05:17

For a pair of oligo length *ℓ* and *k* to cover, we ran Algorithms 2 and 3 to generate an unstructured RNA library covering all *k*-mers in *ℓ*-long sequences. We report the number of oligos in the output of each run and the ratio compared to a theoretical lower bound. Algorithm 2 outputs the incomplete set (oligo length ≤ *ℓ*), and Algorithm 3 outputs the complete set (oligo length = *ℓ*). Reported run times are elapsed times of running Algorithms 2 and 3 consecutively. The naive set is based on generating random sequences until all *k*-mers are covered.

In some cases, no extension forms an unstructured oligo with the current subpath. In these cases, we look for an extension shorter by one nucleotide, and continue shortening until an unstructured path is found or the searched extension is of size 1. This process incurs an additional factor of *O*(*f* (*ℓ*)*cℓ*) per probe in the running time, since in the worst case *ℓ/*2 shortening may occur. Thus, the total running time is *O*(*Xf* (*ℓ*)*cℓ*).

The final result of this process is a set of unstructured probe sequences of length at most *ℓ*. In this set each *k*-mer is represented exactly *p* times. The probes may be of length shorter than *ℓ* in two cases:

1. The path closed a cycle (i.e., reached a vertex with no outgoing edges.)
2. The path had to be shortened to become unstructured, since no unstructured extension was found in *c* attempts.

If the technology requires that all probes have the same length, then an additional process, Algorithm 3, is run to extend these probes into *ℓ*-long unstructured probes. Other methods may be used for this step, such as RNAinverse (Lorenz et al., 2011). The total set in the end is the *complete set*.

If self-structured *k*-mers exist, the algorithm can be used to solve the two subproblems (see section 2.3). The algorithm as is solves the problem of covering all *k*-mers at the expense of having a few structured RNA probes. If structured probes are forbidden, the edges corresponding to self-structured *k*-mers can be removed, and the algorithm can be run on the remaining graph.

**Algorithm 3:** Extend set *S* of RNA sequences covering all *k*-mers, each *p* times, to length *ℓ*. Input: *k* (coverage), *ℓ* (oligo length), *S* (incomplete set), *c* (a limit on the number of attempts)

1: **for** Each *S_i_*∈*S***do**
2: **if** |*S_i_*| = *ℓ***then**
3: Output *S_i_*
4: **else**
5: *attempts* = 0
6: **do**
7: * attempts* = *attempts* + 1
8: Create *ℓ*-long sequence :
9: Pick a random index 1 ≤ *j* ≤ *ℓ* −|*S_i_*| + 1 for *S_i_*.
10: Assign random nucleotides in the positions outside *S_i_*.
11: **while** ( is structured AND (*attempts < c* OR (*attempts <* 100 · *c* AND |*S_i_*| = *k*)))
12: **if** is structured AND |*S_i_*| *> k***then**
13: Continue recursively on the (|*S_i_*|*/*2 + *k/*2)-prefix and (|*S_i_*|*/*2 + *k/*2)-suffix of *S_i_*.
14: Output a union of the returned sets.
15: **else**
16: Output .
17: **end if**
18: ** end if**
19: **end for**

### 3.3. NP-hardness of the minimum *k*-mer coverage by ℓ-long sequences problem

We prove that the following problem is NP-hard: covering all *k*-mers by a minimum-size subset of a restricted set of *ℓ*-long sequences. For the sake of simplicity, we study the problem on the RNA alphabet, but it can be easily generalized to any finite alphabet *Σ*.

The problem is easy in two extreme instances. Clearly, when set *S* contains all possible *ℓ*-long sequences, the problem can be solved in linear time. A de Bruijn sequence can be generated in linear time. Cutting it into *ℓ*-long subsequences with (*k* − 1)-overlaps covers all *k*-mers in the most compact manner. On another extreme, when *ℓ* = *k* the problem is trivial.

We reduce a known NP-hard problem, the minimum *m*-set cover (Levin, 2008), to our problem. While the problems look similar, one is not a private instance of the other and the reduction is not immediate. Here we describe the reduction.

**Theorem 1.** *The minimum k-mer coverage by ℓ-long sequences problem is NP-hard.*

**Proof.** Given an input to the minimum *m*-set cover problem, and a set *S* of subsets of *E* = {*e*_1_…*e_n_*}, we generate an input to the minimum *k*-mer coverage by *ℓ*-long sequences problem in polynomial time. We choose $k = \lceil \log_2 ( n ) \rceil$ and *ℓ* = 3*km*. We map each element *e_i_* ∈ *E* to a *k*-long binary representation of *i*, where instead of bits we use *A* and *U*. We call this representation the element's {*A*, *U*}-representation and denote it by *f_AU_*(*e_i_*).

We generate three sequence sets whose union is the input to the *k*-mer coverage problem.

1. *L*_1_: For each set *S_i_* ∈ *S* we generate a sequence that contains all of its elements' {*A*, *U*}-representation, each buffered by *G^k^* before and *C^k^* after. Formally, for a set *S_i_* = {*e*_*i*_1__,…,*e_i_m__*} we create the sequence: $\prod \nolimits_{j = 1}^{m}{G^k \cdot f_{AU} ( e_{i_j} ) } \cdot C^k$, we append the sequence by *C*'s so that its total length is *ℓ*.
2. *L*_2_: We add sequences that cover all the *k*-mers over {*A*, *U*} that are not covered by *L*_1_. For each *k*-mer *w* over {*A*, *U*} that is not in *L*_1_ we create a sequence *G^k^* · *f_AU_*(*w*) · *C^ℓ^*^−2*k*^.
3. *L*_3_: We cover all non-{*A*, *U*} *k*-mers. Formally, for each *k*-mer $w \in \Sigma^k \backslash \ \{ G^i \{ A , U \} ^{k - i} \cup \{ A , U \} ^j  C^{k - j} \mid 0 \leq i , j \leq k \}$ create the sequence *G^k^* · *w* · *C^ℓ^*^−2*k*^.

The input to the minimum *k*-mer coverage problem is the set *L* = *L*_1_ ∪ *L*_2_ ∪ *L*_3_.

Denote by *L^OPT^* the optimal solution to the *k*-mer coverage problem and by $L_1^{OPT} = L^{OPT} \cap L_1$. The solution to the *m*-set cover problem are the sets corresponding to the sequences in $L_1^{OPT}$. The running time of the reduction is bounded by *O*((4^*k*^ + |*S*|) · *ℓ*) to generate the input sequences, which is *O*((*n*^2^ + |*S*|) · *m* · *log*(*n*)).

We now prove the correctness of the reduction. We start with proving a couple of properties of the solution.

**Lemma 1.** *Any k-mer coverage must include all L*_2_ *sequences.*

**Proof.** Each sequence in *L*_2_ contains a unique *k*-mer over {*A*, *U*}^*k*^ that does not appear in *L*_1_. In addition, by the design of *L*_3_, there are no *k*-mers over {*A*, *U*}^*k*^ in *L*_3_. Thus, to cover all *k*-mers, all of *L*_2_ sequences must be included.   ■

**Lemma 2.** *The selection of sets in L*_3_ *is independent of L*_1_ *and L*_2_.

**Proof.** The set of *k*-mers covered by the selected sequences in *L*_1_ and *L*_2_ is {*G^i^*{*A*, *U*}^*k*-*i*^ ∪ {*A*, *U*}*^j^C*^*k*-*j*^ ∪ *C^g^G^k^*^-*g*^|0 ≤ *i,j,g* ≤ *k*}. The selected sequences in *L*_3_ are constructed to cover all other *k*-mers. It follows that their selection is independent of the input to the problem.   ■

1. *k***-mer coverage** ⇒ *m***-set cover**: All *k*-mers are covered by sequences in *L^OPT^*. The selected sequences from *L*_2_ and *L*_3_ in *L^OPT^* are independent of the input by Lemmas 1 and 2. Each sequence in $L_1^{OPT}$ corresponds to a unique set in *S*. The set of corresponding sets is the optimal solution to the *m*-set cover problem. Assume the contrary, that is, that there exists a smaller solution to the *m*-set cover problem. Then, the set of sequences corresponding to the sets in the solution together with *L^OPT^* ∩{*L*_2_ ∪ *L*_3_} form a smaller solution to the *k*-mer coverage problem, in contradiction to the fact that *L^OPT^* is a minimum *k*-mer coverage.
2. *m***-set cover** ⇒ *k***-mer coverage**: Denote *S^OPT^* to be an optimal solution to the *m*-set cover problem. Denote $L^{ \prime}_1$ as the set of sequences corresponding to the sets in *S^OPT^*. Then, an optimal solution to the *k*-mer coverage problem is the set $L^{ \prime}_3 \cup L_2 \cup L^{ \prime}_1$, where $L^{ \prime}_3$ is the minimum-size set to cover $\Sigma^k \backslash \{ G^i \{ A , U \} ^{k - i} \cup \{ A , U \} ^j C^{k - j} \cup C^g G^{k - g} \mid 0 \leq i , j , g \leq k \}$. All the elements in *E* were covered by *S^OPT^*, and so their {*A*, *U*}-representations are covered by $L^{ \prime}_1$. By Lemmas 1 and 2, *L*_2_ sequences are in any optimal solution, and the selection of *L*_3_ sequences is independent of the input. Assume to the contrary that there exists a smaller solution to the *k*-mer coverage problem. $L_2 \cup L^{ \prime}_3$ are in any solution, so $L^{ \prime}_1$ must be smaller. $L^{ \prime}_1$ covers all the *k*-mers corresponding to the elements in *E*, so there is a smaller solution to the *m*-set cover problem, in contradiction to the fact that *S^OPT^* is an optimal solution.   ■

Clearly, if we could solve the *p*-multi *k*-mer coverage problem in polynomial time, then we could solve the *k*-mer coverage problem. Thus, we get:

**Corollary 2.** *The minimum p-multi k-mer coverage by ℓ-long sequences is NP-hard.*

## 4. Results

### 4.1. Traditional methods won't solve our problem

**FIG. 2.**
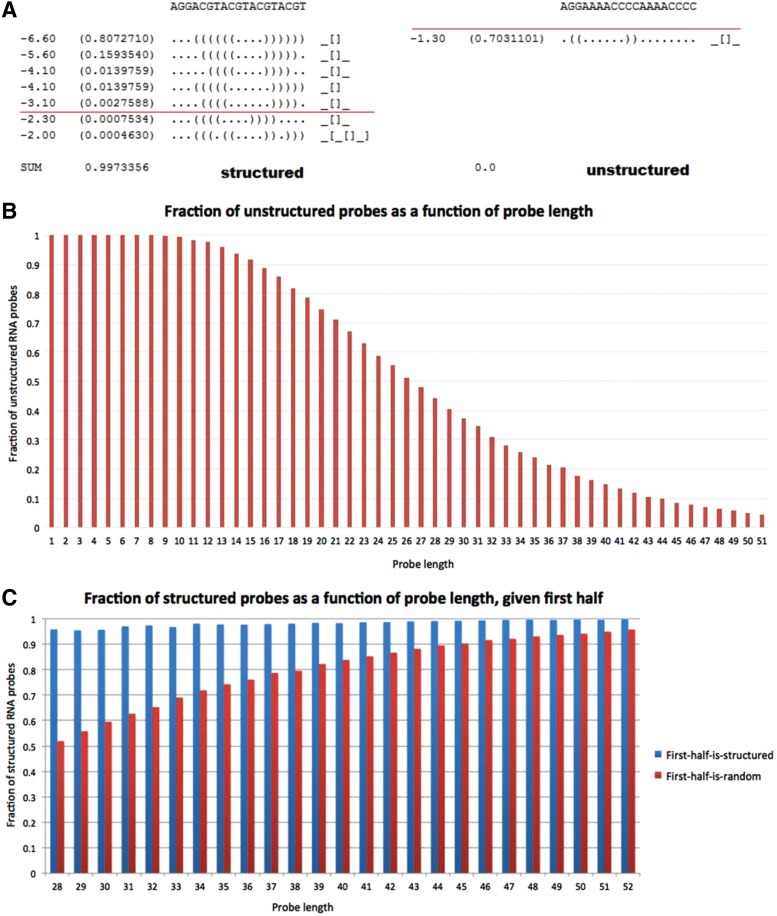
Properties of unstructured oligos. **(A)** An output of RNAshapes. For each probe sequence, an ensemble of structures is predicted. If the sum of probabilities of structures with energy smaller than −2.5 is greater than 0.5, the oligo is considered *structured*. On the left is a highly structured oligo, while on the right an unstructured oligo. **(B)** Fraction of unstructured RNA probes as a function of their length. For each probe length, the fraction of unstructured RNA probes was empirically estimated using 10,000 randomly generated sequences of this length. **(C)** Fraction of structured RNA probes as a function of their length given their first half. For each probe length, the fraction of structured RNA probes was empirically estimated using 100 structured (blue)/random (red) first halves. For each first half, 100 random extensions were appended to generate a complete probe.

We sought to test whether traditional methods to cover all *k*-mers, such as random oligos or overlapping subsequences of a de Bruijn graph, could solve the minimum *k*-mer coverage problem. Toward this aim, we analyzed the properties of unstructured probe sequences. Here we followed the definition used in the RNAcompete study (Ray et al. 2009). Predicting a single minimum folding energy structure may be misleading, as many RNAs may fold into different structures. Thus, for each RNA sequence an ensemble of structures is predicted. The oligo is considered structured if its probability of forming a low energy structure is more than half. Figure 2A depicts the structuredness test. Unfortunately, this property cannot be elegantly formulated in combinatorial terms. For a formal definition and technical details, see section 2.2.

To better understand the problem at hand, we calculated the percentage of unstructured RNA probes. Ideally, we would iterate over all possible RNA *ℓ*-long sequences and test if each is structured. While for small values of *ℓ* this strategy is feasible, for greater values it is not, as it requires 4^*ℓ*^ iterations. To overcome this problem, we generated 10,000 random *ℓ*-long sequences for each value of *ℓ*, where each nucleotide is uniformly picked at each position in the sequence. The fraction of structured probes quickly converged (data not shown), and hence we are confident that these estimates are accurate.

The results are shown in Figure 2B. As expected, the fraction of structured probes is higher for longer probes. More surprisingly, the decrease in the fraction of unstructured RNA probes as a function of length is fast, and for length 45, less than 10% of the probes are unstructured. Thus, using random oligos is suboptimal and requires many more probe sequences to cover all *k*-mers. De Bruijn sequences, which are the most compact sequences to cover all *k*-mers, are uniformly distributed over all *k*-mers (MacWilliams and Sloane, 1976) and are therefore prone to having many structured subsequences. Indeed, in the report of the RNAcompete study (Ray et al., 2009), in a de Bruijn sequence of order 11 over {*A*, *C*, *G*, *U*}, only 36,837 probes out of 167,773 were unstructured. Note also that for *k* ≥ 9 the fraction is smaller than 1 due to self-structured *k*-mers (see section 2.2). To conclude, neither random oligos nor probes generated by overlapping subsequences of a de Bruijn sequence are likely to provide an optimal or near-optimal solution to our problem.

To support our assumption that structured subsequences are likely to be extended to structured sequences (see section 3.2), we calculated the fraction of structured probes given that their first half is structured. For each probe length 28 ≤ *ℓ* ≤ 52, we generated 100 random $\lfloor \ell / 2 \rfloor$-long structured sequences and extended them by 100 random extensions to a probe of length *ℓ*. The fraction of structured probes out of the 10,000 probes is the reported value. We compared this value to the fraction of structured probes among random *ℓ*-long sequences. Results show that, given that the first half is structured, there is a chance of more than 95% that a probe starting with it is structured (see Fig. 2C). The fraction of structured oligos among random oligos is much smaller, supporting our assumption that structured subsequences are more likely to be extended to structured sequences.

### 4.2. A theoretical lower bound for the number of oligos

We give a simple lower bound for the number of oligos needed to cover all *k*-mers based on *k*-mer counts. Since we do not know the optimal solution to the theoretical problem, we will use this lower bound as a baseline to compare to.

Denote the minimum number of oligos to be *n*(*k*, *ℓ*), where *k* is the desired *k*-mer coverage and *ℓ* the length of the probe. Then,

\begin{align*}n ( k , \ell ) \geq \lceil \frac { 4^k }  { \ell - k + 1 } \rceil \tag { 1 } \end{align*}

It follows immediately from the fact that the number of *k*-mers to be covered is 4^*k*^ and each probe of length *ℓ* covers *ℓ* − *k* + 1 *k*-mers.

For the *p*-multi *k*-mer coverage, the bound is:

\begin{align*}n ( k , \ell , p ) \geq \lceil \frac { 4^k \cdot p }  { \ell - k + 1 } \rceil \tag { 2 } \end{align*}

### 4.3. Computational results

We implemented and ran our heuristic algorithm on 5 ≤ *k* ≤ 10 and *ℓ* = 30,35,40, typical values used for library design (Ray et al., 2009; Kierzek et al., 2015). Multiplicity was set to 1, number of random attempts to 100, and randomization seed to 0. The results are summarized in Table 1. On average, our method generates a library that is only 1.1–1.3 times greater in size than the theoretical lower bound. Moreover, as expected, the ratio compared to the lower bound increases with oligo length. It is more difficult to find unstructured probes since the fraction of unstructured probes decreases with oligo length (see section 4.1). Note that for *k* ≥ 9, there are a few structured probes in the set. These cannot be avoided due to self-structured *k*-mers (see section 4.1). Running times were benchmarked on a single CPU of a 20-CPU Intel Xeon E5-2650 (2.3GHz) machine with 384GB 2133MHz RAM.

In addition, we implemented a naive algorithm to compare the performance with our algorithm. We generated random sequences of length *ℓ* and added them if they included uncovered *k*-mers until all *k*-mers were covered. We report the average set size over 100 runs. As can also be seen in Table 1, the naive algorithm produces much larger sets than our heuristic.

### 4.4. Comparison to the library design of Ray *et al*.

To compare our solution to the library design of RNAcompete (Ray et al., 2013), we ran the algorithm with *k* = 9, *ℓ* = 35, and *p* = 16, as their library is required to cover each 9-mer at least 16 times. Notably, our solution is significantly more compact. Our library contains a total of 166,649 oligos of length 35. Compared to the theoretical lower bound of 155,346 oligos, our library is only 1.07 times greater in size. In comparison, the library of Ray et al. contains 214,948 probes, which is 1.38 times greater in size than the theoretical lower bound. Moreover, in our complete library, all oligos have the same length, as opposed to the library of Ray et al., where oligo lengths vary. A more flexible length requirement may enable us to construct an even smaller library. More importantly, their library includes 2,858 structured probes due to self-structured 9-mers, while in our library there are only 841, a very small fraction of the total number of probes.

## 5. Conclusion

In this work, we have presented, for the first time, a general algorithm to generate a compact set of unstructured RNA probes that together cover all RNA *k*-mers. The algorithm's good performance can be attributed to the key ideas of generating probe sequences using de Bruijn graphs, but taking a random walk on those and backtracking when we encounter a structured sequence.

De Bruijn graphs and linear-feedback shift registers (LFSR) are commonly used to generate de Bruijn sequences. Euler tours over de Bruijn graphs have the advantage that all possible (4!)^4^*k*−1^^*/*4^*k*^ de Bruijn sequences can be generated (Bruijn, 1946). On the other hand, linear-shift feedback registers for generating de Bruijn sequences are limited by the number of primitive polynomials over *GF*(4) with degree *k* (Lempel, 1970). There are only *φ*(4^*k*−1^)*/k* primitive polynomials, where *φ* is the totient function. For example, for *k* = 11 there are only 240,064 de Bruijn sequences that can be generated by an LFSR. In addition, LFSR-generated sequences have uniform properties (Hurd, 1974), which are counter-productive to the problem at hand, since it requires local properties of unstructuredness. Thus, de Bruijn graphs provide a much more flexible mechanism than LFSRs to generate sets of sequences with specific properties covering all *k*-mers.

Our implementation deals cleverly with prohibitive running times. Our backtracking approach is particularly suited to the monotone property of RNA secondary structure. That is, having a structured subsequence highly influences the probability of the whole sequence being structured. In addition, the random walk works in a way that tries to double the length of the path in each attempt, and in so doing reduces the running time of the extension process by a factor of *ℓ*. We applied several practical heuristics, such as a limited number of attempts and shortening extensions, to avoid dead-end paths.

The potential downside of our approach is its heuristic nature, which intrinsically does not guarantee any ratio over the optimal solution. Unfortunately, the structuredness property of RNA sequences is not easily translated into combinatorial properties that can be targeted by short paths in a de Bruijn graph. Properties that proximate these features, such as not having a *k*-mer and its reverse complement in the same probe sequence, are not good enough to ensure that the probe is unstructured by the prediction algorithm.

While in this work we focused on one application, we see the substantial potential benefit of our algorithm in other applications. Our general scheme can be used to design sequence libraries with other desired properties or other definitions of structuredness. For example, RNA secondary structure can be defined by minimum free-energy instead of an ensemble of structures (Churkin et al., 2015). On the DNA front, DNA oligos with specific DNA shape features are desirable as shape plays a significant role in protein DNA-binding (Burgess, 2015). Moreover, our algorithm can be modified to cover only a subset of the *k*-mers, or have different multiplicities for each *k*-mer, by keeping the edges in the de Bruijn graph that represent those *k*-mers and add different numbers of edge copies for each *k*-mer. For example, in the RNAcompete technology two 7-mers are excluded as they are restriction sites of an enzyme used in the protocol (Ray et al., 2009).

To conclude, we have demonstrated the ability of our algorithm to meet the highly desired goal of generating compact sets of unstructured RNA probes that cover all *k*-mers. High-throughput technologies that measure RNA accessibility as part of the secondary structure or protein RNA-binding *in vitro* will greatly benefit from this design. The generated library set is only slightly larger than the theoretical lower bound, and thus achieves near-optimal results. The algorithms can be easily applied to other sequence design problems. Any design that requires complete coverage of all *k*-mers, with specific sequence properties, can utilize our general scheme of random path search in de Bruijn graphs.

## References

[B1] Alhakim A., and Akinwande M. 2011 A recursive construction of nonbinary de Bruijn sequences. Des. Codes Cryptogr. 60, 155–169

[B2] Berger B., Peng J., and Singh M. 2013 Computational solutions for omics data. Nat. Rev. Genet. 14, 333–346 23594911 10.1038/nrg3433 PMC3966295

[B3] Berger M.F., Philippakis A.A., Qureshi A. M., et al. 2006 Compact, universal DNA microarrays to comprehensively determine transcription-factor binding site specificities. Nat. Biotechnol. 24, 1429–1435 16998473 10.1038/nbt1246 PMC4419707

[B4] Berman P., DasGupta B., and Sontag E. 2004 Randomized approximation algorithms for set multicover problems with applications to reverse engineering of protein and gene networks, 39–50. In Jansen K., Khanna S., Rolim J.D.P., and Ron D., eds. Approximation, Randomization, and Combinatorial Optimization. Algorithms and Techniques. Springer, Cambridge, MA

[B5] Burgess D.J. 2015 DNA elements: Shaping up transcription factor binding. Nat. Rev. Genet. 16, 258–259 25446317 10.1038/nrg3874

[B6] Churkin A., Weinbrand L., and Barash D. 2015 Free energy minimization to predict RNA secondary structures and computational RNA design. Methods Mol. Biol. 1269, 3–16 25577369 10.1007/978-1-4939-2291-8_1

[B7] de Bruijn N. 1946 A combinatorial problem. Proc. Kon. Ned. Akad. Wet. Ser. A 49, 758

[B8] Fordyce P.M., Gerber D., Tran D., et al. 2010 *De novo* identification and biophysical characterization of transcription-factor binding sites with microuidic affinity analysis. Nat. Biotechnol. 28, 970–975 20802496 10.1038/nbt.1675 PMC2937095

[B9] Fu X.-D., and Ares M. Jr., 2014 Context-dependent control of alternative splicing by RNA-binding proteins. Nat. Rev. Genet. 15, 689–701 25112293 10.1038/nrg3778 PMC4440546

[B10] Gerstberger S., Hafner M., and Tuschl T. 2014 A census of human RNA-binding proteins. Nat. Rev. Genet. 15, 829–845 25365966 10.1038/nrg3813 PMC11148870

[B11] Grossman T., and Wool A. 1997 Computational experience with approximation algorithms for the set covering problem. Eur. J. Oper. Res. 101, 81–92

[B12] Hurd W.J. 1974 Efficient generation of statistically good pseudonoise by linearly interconnected shift registers. IEEE Trans. Comput. 100, 146–152

[B13] Kertesz M., Iovino N., Unnerstall U., et al. 2007 The role of site accessibility in microRNA target recognition. Nat. Genet. 39, 1278–1284 17893677 10.1038/ng2135

[B14] Kertesz M., Wan Y., Mazor E., et al. 2010 Genome-wide measurement of RNA secondary structure in yeast. Nature 467, 103–107 20811459 10.1038/nature09322 PMC3847670

[B15] Kierzek E., Kierzek R., Turner D.H., et al. 2006 Facilitating RNA structure prediction with microarrays. Biochemistry 45, 581–593 16401087 10.1021/bi051409+ PMC4070881

[B16] Kierzek R., Turner D.H., and Kierzek E. 2015 Microarrays for identifying binding sites and probing structure of RNAs. Nucleic Acids Res. 43, 1–12 25505162 10.1093/nar/gku1303 PMC4288193

[B17] König J., Zarnack K., Luscombe N.M., et al. 2012 Protein–RNA interactions: New genomic technologies and perspectives. Nat. Rev. Genet. 13, 77–83 22251872 10.1038/nrg3141

[B18] Kudla G., Granneman S., Hahn D., et al. 2011 Cross-linking, ligation, and sequencing of hybrids reveals RNA–RNA interactions in yeast. Proc. Natl. Acad. Sci. U. S. A. 108, 10010–10015 21610164 10.1073/pnas.1017386108 PMC3116431

[B19] Lambert N., Robertson A., Jangi M., et al. 2014 RNA bind-n-seq: Quantitative assessment of the sequence and structural binding specificity of RNA binding proteins. Mol. Cell 54, 887–900 24837674 10.1016/j.molcel.2014.04.016 PMC4142047

[B20] Lempel A. 1970 On a homomorphism of the de Bruijn graph and its applications to the design of feedback shift registers. IEEE Trans. Comput. 100, 1204–1209

[B21] Levin A. 2008 Approximating the unweighted k-set cover problem: Greedy meets local search. SIAM J. Discrete Math. 23, 251–264

[B22] Lorenz R., Bernhart S.H., Zu Siederdissen C.H., et al. 2011 ViennaRNA package 2.0. Algorithms Mol. Biol. 6, 26 22115189 10.1186/1748-7188-6-26 PMC3319429

[B23] MacWilliams F.J., and Sloane N.J. 1976 Pseudo-random sequences and arrays. Proc. IEEE 64, 1715–1729

[B24] Mandir J.B., Lockett M.R., Phillips M.F., et al. 2009 Rapid determination of RNA accessible sites by surface plasmon resonance detection of hybridization to DNA arrays. Anal. Chem. 81, 8949–8956 19874056 10.1021/ac9015962 PMC2771317

[B25] Orenstein Y., and Shamir R. 2013 Design of shortest double-stranded DNA sequences covering all k-mers with applications to protein-binding microarrays and synthetic enhancers. Bioinformatics 29, i71–i79 23813011 10.1093/bioinformatics/btt230 PMC3694677

[B26] Philippakis A.A., Qureshi A.M., Berger M.F., et al. 2008 Design of compact, universal DNA microarrays for protein binding microarray experiments. J. Comput. Biol. 15, 655–665 18651798 10.1089/cmb.2007.0114 PMC3203512

[B27] Ray D., Kazan H., Chan E.T., et al. 2009 Rapid and systematic analysis of the RNA recognition specificities of RNA-binding proteins. Nat. Biotechnol. 27, 667–670 19561594 10.1038/nbt.1550

[B28] Ray D., Kazan H., Cook K.B., et al. 2013 A compendium of RNA-binding motifs for decoding gene regulation. Nature 499, 172–177 23846655 10.1038/nature12311 PMC3929597

[B29] Rinn J.L., and Ule J. 2014 Oming in on RNA–protein interactions. Genome Biol. 15, 401 24485348 10.1186/gb4158 PMC4053986

[B30] Steffen P., Voss B., Rehmsmeier M. et al. 2006 RNAshapes: An integrated RNA analysis package based on abstract shapes. Bioinformatics 22, 500–503 16357029 10.1093/bioinformatics/btk010

[B31] Stefl R., Skrisovska L., and Allain F.H.-T. 2005 RNA sequence-and shape-dependent recognition by proteins in the ribonucleoprotein particle. EMBO Rep. 6, 33–38 15643449 10.1038/sj.embor.7400325 PMC1299235

[B32] Wan Y., Kertesz M., Spitale R.C., et al. 2011 Understanding the transcriptome through RNA structure. Nat. Rev. Genet. 12, 641–655 21850044 10.1038/nrg3049 PMC3858389

[B33] West D.B., et al. 2001 Introduction to Graph Theory, Vol. 2 Prentice Hall, Upper Saddle River, NJ

